# Correction: Development of a structure-based computational simulation to optimize the blocking efficacy of pro-antibodies

**DOI:** 10.1039/d1sc90153b

**Published:** 2021-07-20

**Authors:** Bo-Cheng Huang, Yun-Chi Lu, Jun-Min Liao, Hui-Ju Liu, Shih-Ting Hong, Yuan-Chin Hsieh, Chih-Hung Chuang, Huei-Jen Chen, Tzu-Yi Liao, Kai-Wen Ho, Yeng-Tseng Wang, Tian-Lu Cheng

**Affiliations:** Institute of Biomedical Sciences, National Sun Yat-Sen University Kaohsiung Taiwan tlcheng@kmu.edu.tw; Department of Biomedical Science and Environmental Biology, Kaohsiung Medical University Kaohsiung Taiwan; Drug Development and Value Creation Research Center, Kaohsiung Medical University Kaohsiung Taiwan; Graduate Institute of Medicine, College of Medicine, Kaohsiung Medical University Kaohsiung Taiwan; School of Medicine for International Students, I-Shou University Kaohsiung Taiwan; Department of Medical Laboratory Science and Biotechnology, College of Health Sciences, Kaohsiung Medical University Kaohsiung Taiwan; Department of Medical Research, Kaohsiung Medical University Hospital Kaohsiung Taiwan; Department of Biochemistry, Kaohsiung Medical University Kaohsiung Taiwan c00jsw00@kmu.edu.tw

## Abstract

Correction for ‘Development of a structure-based computational simulation to optimize the blocking efficacy of pro-antibodies’ by Bo-Cheng Huang *et al.*, *Chem. Sci.*, 2021, DOI: 10.1039/D1SC01748A.

The authors regret that a funding source was omitted from the original article. The following funding information should have been acknowledged: Ministry of Science and Technology, Taiwan (MOST 109-2627-M-037-001) and Kaohsiung Medical University, Taiwan (KMU-DK(B)11000-4).

The Royal Society of Chemistry apologises for these errors and any consequent inconvenience to authors and readers.

## Supplementary Material

